# In Vitro Adhesion and Invasion Rates of *Staphylococcus aureus* Isolated from Mastitic Cows Are Modulated by the *agr* System and *MSCRAMM* Genes

**DOI:** 10.3390/vetsci12030270

**Published:** 2025-03-13

**Authors:** Erika Carolina Romão Bonsaglia, Bruna Fernanda Rossi, Fabio Sossai Possebon, Nathalia Cristina Cirone Silva, Juliano Leonel Gonçalves, Ivana Giovannetti Castilho, Ary Fernandes Junior, Marcos Veiga dos Santos, Vera Lúcia Mores Rall

**Affiliations:** 1 Department of Animal Nutrition and Production, School of Veterinary Medicine and Animal Sciences, University of São Paulo (USP), Pirassununga 13635-900, Brazil; mveiga@usp.br; 2 Department of Chemical and Biological Sciences, Institute of Biosciences, Sao Paulo State University, Botucatu 18618-691, Brazil; brunabiounesp@gmail.com (B.F.R.); ivancas02@yahoo.com.br (I.G.C.); ary.fernandes@unesp.br (A.F.J.); vera.rall@unesp.br (V.L.M.R.); 3 Department of Animal Production and Preventive Veterinary Medicine, Sao Paulo State University, Botucatu 18618-691, Brazil; fabio.possebon@unesp.br; 4 Institute of Biotechnology, Sao Paulo State University (UNESP), Botucatu 18618-691, Brazil; 5 Department of Food Science and Nutrition, School of Food Engineering, University of Campinas (UNICAMP), Campinas 13083-862, Brazil; ncirone@unicamp.br; 6 Bacteriology, Veterinary Diagnostic Laboratory, College of Veterinary Medicine, Michigan State University, Lansing, MI 48910, USA; goncal25@msu.edu

**Keywords:** dairy cow, pathogenicity, mastitis severity, clinical mastitis, subclinical mastitis

## Abstract

Mastitis, an inflammatory condition of the udder, can be caused by the entry of *Staphylococcus aureus*, whose adhesion to the mammary epithelial cells is influenced by virulence factors such as microbial surface components recognizing adhesive matrix molecules (MSCRAMMs) and the accessory gene regulator (*agr*) system. Our goal was to determine the adhesion and invasion rates of *S. aureus* isolates from clinical (mild and moderate) and subclinical mastitis and to assess the impact of MSCRAMM genes and *agr* types on disease severity. Clinical isolates predominantly carried *agrII* (*p* < 0.0083) and multiple MSCRAMM genes, correlating with high adhesion capacity but reduced invasion capacity regardless of clinical severity. Remarkably, subclinical isolates, mainly *agr*-negative (85.7%), showed increased cellular invasion (*p* < 0.0001), possibly due to reduced expression of *agr*-mediated virulence factors. These findings contribute to the understanding of the pathogen–host dynamics in bovine mastitis and highlight the importance of both MSCRAMMs and the *agr* system in modulating disease severity. These insights can inform targeted interventions for mastitis prevention and treatment.

## Simple Summary

Mastitis, an inflammatory condition of the udder, often involves *Staphylococcus aureus*, whose adhesion to mammary epithelial cells is influenced by virulence factors like MSCRAMM genes and the *agr* system. This study analyzed adhesion and invasion rates of *S. aureus* from clinical and subclinical mastitis cases, linking these traits to MSCRAMM and *agr* types. Clinical isolates, predominantly *agrII*-positive, exhibited high adhesion but limited invasion. Subclinical isolates, mostly *agr-*negative, showed increased invasion, likely due to reduced *agr*-mediated virulence. These findings enhance the understanding of pathogen–host interactions in mastitis and support the development of targeted strategies for prevention and treatment.

## 1. Introduction

Mastitis, an inflammatory condition of the udder, can manifest clinically or subclinically. *Staphylococcus aureus* is a known causative agent of both forms of mastitis [1]. The resulting damage caused to mammary tissue leads to decreased milk production and altered milk composition. This occurs due to the interaction between bacteria and host cells, a hallmark of disease development. *S. aureus* utilizes Microbial Surface Components Recognizing Adhesive Matrix Molecules (MSCRAMMs) proteins to adhere to host tissues and establish infection [2]. MSCRAMM proteins play a crucial role by adhering to specific host extracellular components, aiding in colonization, and evading the host’s immune system [2]. Fibrinogen-binding proteins (*fib*) and clumping factors (*clfA*, *clfB*) promote bacterial adherence and aggregation, while fibronectin-binding proteins (FnBPs) facilitate binding to fibronectin, fibrinogen, and elastin, promoting the internalization of *S. aureus* into epithelial and endothelial cells that are normally non-phagocytic. Both FnBPs A and B are encoded by two closely linked but separately transcribed genes, *fbnA* and *fbnB* [3]. Adhesion to fibronectin is a critical step in establishing the pathogenesis of bovine mastitis [4]. Collagen-binding protein (*cna*) mediates collagen binding. These interactions not only increase bacterial adherence but also contribute to the pathogenesis of *S. aureus* infections, promoting invasion into deeper tissue layers and ultimately making antibiotic treatment difficult due to the bacteria’s ability to evade the immune system and establish intracellular reservoirs [5,6]

The invasive potential of microorganisms is a key virulence factor in mastitis, as it facilitates survival and replication within epithelial cells, evasion of host defenses, and long-term persistence in the host without causing visible inflammation [7]. The *agr* system, a molecular typing method used to assess the phylogenetic relationship among *S. aureus* isolates, regulates the expression of numerous virulence factors.

The accessory gene regulator (*agr*) is a quorum-sensing-associated system that contributes to host infection [8] by regulating virulence factors, such as cell wall-associated components and extracellular toxins [9] According to Yang et al. [10], *agr* mediates the transition from the expression of *S. aureus* adhesion and colonization factors at low cell densities to the expression of toxins and extracellular enzymes at high cell densities. *S. aureus* strains encode one of four *agr* variants (I–IV), and certain *agr* groups may be associated with invasive behavior, providing significant insights into the virulence and pathogenicity of these isolates [11].

The aim of this study is to investigate the differences in the cellular adhesion and invasion rates of *S. aureus* isolates from different clinical bovine mastitis severities and to correlate these with *agr* types and MSCRAMM genes.

## 2. Materials and Methods

### 2.1. S. aureus Isolates and Genes Assessed

A total of 42 isolates of *S. aureus* were utilized in this study. These isolates included 14 from moderate clinical mastitis, 14 from mild clinical mastitis, and 14 from subclinical mastitis. All isolates are part of the bacterial collection maintained by the Milk Quality Research Laboratory (Qualileite Lab., Pirassununga, Brazil) at the University of São Paulo, Brazil and clinical isolates were collected in previous studies [12] from 20 dairy herds from southeastern Brazil (15 from the State of São Paulo and 5 from the State of Minas Gerais). The minimum number of isolates per type of mastitis or severity was selected to meet budget requirements. The study was approved by the Ethics Committee on Animal Use of the School of Veterinary Medicine and Animal Science of University of São Paulo (registration code: CEUA 2994060214).

The presence of *agr genes* (*I*, *II*, *III*, *IV*) and several MMSCRAM genes (*fnbA*, *fnbB*, *fib*, *clfA*, *clfB*, *cna*, *eno*, and *epbS*) was investigated using PCR assays, according to Supplementary Table S1. Bacterial DNA extraction was performed using the Illustra Bacteria Mini Spin Kit (GE Healthcare, Buckinghamshire, UK) following the manufacturer’s instructions. The nuclease gene (*nuc*) was amplified for species confirmation [9,13].

### 2.2. Adhesion and Invasion Assays

Adhesion assay: Isolates were cultured in tryptic soy broth (TSB, Oxoid, Basingstoke, UK) at 37 °C until reaching the stationary growth phase, and then diluted in DMEM (Sigma-Aldrich, St. Louis, MO, USA) to achieve a suspension of approximately 1.5 × 10^7^ CFU/mL. Bovine mammary epithelial cells (MAC-T) cells were cultured in 24-well plates. Wells were rinsed three times with PBS+ buffer (0.01 M PBS, supplemented with 0.1 g/L CaCl_2_ and 0.2 g/L MgCl_2_; Sigma-Aldrich), before adding 1 mL the bacterial suspension. After 3 h of incubation, non-adherent bacteria were removed by washing. MAC-T were resuspended in 500 µL trypsin–EDTA solution (0.1%/0.04%), homogenized, and serial dilutions in saline solutions were performed. Then, 10 µL were plated onto tryptic soy agar (TSA, Oxoid, Basingstoke, UK), and incubated at 35 °C for 24 h to confirm bacterial recovery. Both adhesion and invasion assays were performed in triplicate in 24-well plates (per assay/strain), and the experiments were independently replicated three times to ensure the reliability of the results [14].

Invasion assay: All conditions were maintained as previously described until the initial three hours of incubation were completed. After washing, cells were treated with Eagle’s solution containing 200 µg/mL gentamicin (Sigma-Aldrich, St. Louis, MO, USA) and incubated for 2 h, to eliminate the extracellular bacteria. The absence of growth on TSA agar confirmed the effectiveness of gentamicin. Cells were lysed with 0.1% Triton X-100 (Sigma-Aldrich, St. Louis, MO, USA), diluted, plated onto TSA plates, and incubated. The invasion percentage was calculated based on recovered colonies compared to adhesion assay values (adhered bacteria/internalized bacteria × 100) [14].

### 2.3. Statistical Analysis

The frequency of each *agr* type and MSCRAMM genes was calculated as the proportion of positive isolates for each gene divided by the total number of isolates in the clinical and subclinical categories. Chi-square or Fisher Exact tests (PROC FREQ; SAS Institute, Cary, NC, USA) were used, with a significance level of 0.05.

Adhesion and invasion cell counts were log-transformed for analysis. Subsequently, the ratio of adhesion to invasion was calculated by dividing the log of adhesion counts by the log of invasion counts. The normality of the response variables was assessed using both statistical tests and graphical analysis. Differences in the medians between clinical severity groups were conducted using the Kruskal–Wallis test followed by Dunn’s post-hoc analysis. Statistical significance was considered when *p* < 0.05. A correlation of adhesion and invasion genes was obtained using the Mann–Whitney test. Statistical analysis and graphical representation were generated using GraphPad Prism version 8.0.1.

## 3. Results

### 3.1. Distribution of Genes in S. aureus Isolates

Table 1 shows the distribution of *agr* types and MSCRAMM genes among *S. aureus* isolates. The *agrII* gene (*p* < 0.0014) was more prevalent in cases of clinical mastitis than in subclinical. The *agrIV* gene was not found in any isolates. Conversely, the *agr*-negative (*agr*-) isolates predominate in subclinical cases (*p* < 0.00002). Genes *fnbA* (*p* < 0.0001), *fib* (*p* < 0.000000007), and *ebps* (*p* < 0.0001) were more prevalent in isolates from mild and moderate clinical cases compared to subclinical cases. The *eno* gene was prevalent in all groups.

### 3.2. Correlation Between Adhesion and Invasion Capacity with Clinical Mastitis Severitiy

**Figure 1:**
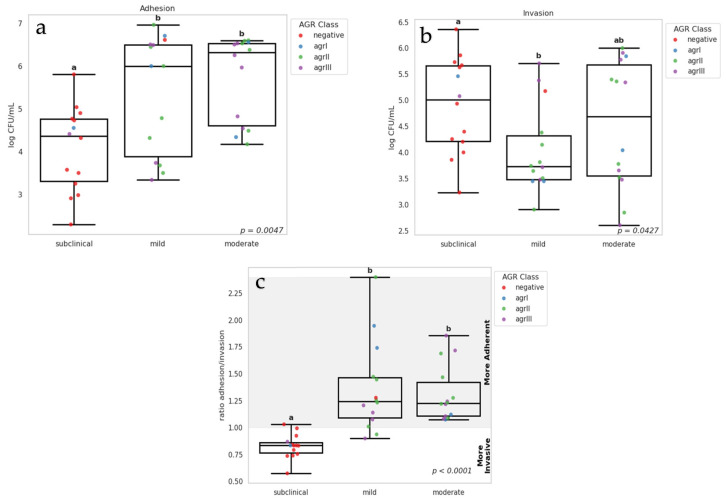
Box Plots showing adhesion (**a**) and invasion (**b**) capacity of *S. aureus* isolates to MAC-T cells. Box plot (**c**) illustrates the ratio between adhesion and invasion. Isolates with values lower than 1 are considered more invasive, while values higher than 1 indicate higher adhesion capacity. Individual samples are indicated in dots with different colors for each *agr* class. Different letters indicate significant differences between subclinical and clinical (mild and moderate). *p* value from Kruskal–Wallis test.

Figure 1a,b shows a comparative analysis of adhesion and invasion rate differences between subclinical and clinical isolates (*p* = 0.0047), with clinical isolates exhibiting higher adhesion levels than subclinical. However, among clinical isolates (mild and moderate), no difference was observed in adhesion capacity. Regarding invasion, subclinical isolates had higher invasion capacity than mild clinical isolates. Conversely, moderate clinical isolates exhibited a high variation in invasion capacity, showing no difference when compared to subclinical and mild clinical isolates (*p* = 0.0427). We also analyzed the ratio between adhesion and invasion (Figure 1c), and observed that subclinical mastitis isolates had values ≤ 1; therefore, we considered these isolates as more invasive. On the other hand, the isolates from clinical mastitis (mild and moderate) that presented values > 1 and were considered more adherent.

In our study, we observed in Table 2 a significant correlation between *agr* genes and cellular adhesion/invasion ratio. Specifically, the absence of the *agr* gene was associated with lower cellular adhesion rate (*p* < 0.004), indicating that the presence of *agr* is crucial in this process, irrespective of clinical severity. On the other hand, *agrII* isolates demonstrated higher adhesion/invasion ratio compared to other *agr* types. When analyzing the adhesion/invasion ratio, we found that *agr*-negative isolates showed greater cellular invasion capacity. The frequency of MMSCRAM genes*, fnbA*, *clfA*, and *ebpS* showed no correlation in adhesion or invasion assay. However, when comparing adhesion/invasion rates, isolates positive for these genes tended to exhibit higher adhesion capacity (see Figure 1c). The absence of the *fnbB* gene was associated with adhesion, with no significant differences in adhesion/invasion rates. Conversely, the presence of the *fib* gene was statistically associated with cellular adhesion, confirmed by the adhesion/invasion ratio, where these isolates demonstrated greater adhesion capacity (1.23) than invasion.

## 4. Discussion

The onset of mastitis is marked by the entry of *Staphylococcus aureus* into the mammary gland and its adherence to epithelial cells. The success of the infection depends on *S. aureus*’s ability to evade host defenses and invade mammary tissue, facilitated by virulence factors, such as MSCRAMMs and the *agr* system [15]. MSCRAMMs and the *agr* system are mechanisms that mediate bacterial adherence, consequently favoring their survival and pathogenicity and potentially influencing disease severity. Our results showed that the presence of the *fnbA* gene is associated with a higher adherence capacity. This is consistent with previous findings demonstrating reduced colonization ability in FnBP-deficient strains by Brouillette et al. [16]. Furthermore, the overexpression of *fnb* genes results in increased invasion of bovine mammary epithelial cells (BMEC) [17]. In our study, the highest adherence rate was observed in clinical isolates, all of which were *agr* positive, which corroborates with the negative regulation of MSCRAMM expression by *agr* [18]. The expression of FnBPs is also associated with increased invasiveness of isolates [19], also corroborating our results, where the absence of this gene was associated solely with adherence. However, the presence of *fnbA* and *fnbB* genes is not an essential factor for cellular adherence, as bacteria can still invade even in the absence of both genes [20], as demonstrated in our study, where subclinical isolates were able to invade even in the absence of these genes.

The presence of *fib*, *clfA*, and *ebpS* genes was also associated with adherence. We observed high prevalence of these genes in clinical isolates, which carried at least four MSCRAMM genes. In contrast, subclinical isolates predominantly presented one MSCRAMM gene (*eno*). However, there is redundancy in MSCRAMMs as some of them can bind to multiple host proteins, and likewise, several host proteins can bind to the same MSCRAMM [21]. We suggest that the presence of a minimum number of genes may facilitate cellular invasion, while a greater number of these genes may prolong cell adherence for a longer time, resulting in increased activation of the immune system and clinical signs of the disease.

In a previous study, we showed the prevalence of *agr* type I or *agr*-negative in subclinical isolates of *S. aureus* [9]. In this study, the highest cellular invasion rate was observed in cases of subclinical mastitis, where isolates were predominantly *agr*-negative. Host cell invasion is linked to *S. aureus*’s ability to persist in the intracellular environment for long periods. According to Siegmund et al. [22], the intracellular adaptation of *S. aureus* is due to phenotypic transition to small colony variants, which are characterized by slow growth and reduced metabolism rates. Additionally, the reduction in virulence factor expression, mediated by the absence of *agr*, facilitates evasion of the immune response.

Our results indicated that the most adherent isolates were from clinical mastitis (mild and moderate) and were *agr* positive, mainly *agrII*, which was associated with adherence. Isolates carrying these genes are more commonly associated with clinical mastitis [20]. While *agr* negatively regulates the expression of MSCRAMMs [18], *agr*-positive isolates can persist or coexist in an infection with *agr*-negative isolates, where they benefit from the initial adherence and colonization of *agr*-negative isolates [23]. Furthermore, *agr* positively regulates the expression of extracellular toxins (hemolysins, enterotoxins, extracellular proteases, etc.), which justify clinical signs of the disease [18]. These findings reinforce the role of *agr* and MSCRAMM genes in modulating *S. aureus* pathogenicity and may have important implications for mastitis control strategies. The relationship between adhesion and invasion is complex and not always directly correlated. While adhesion is often a prerequisite for invasion, a strain that adheres well may lack the additional factors required for active invasion, such as the ability to manipulate host cell pathways or evade immune responses. Previous studies show that adhesion and invasion can be regulated by different genes and influenced by environmental factors [24,25]. Our results suggest that isolates from clinical mastitis, predominantly *agr* II-positive and carrying multiple MSCRAMM genes, exhibit strong adhesion but limited invasion, which may prolong bacterial persistence in the mammary gland and contribute to sustained immune activation. In contrast, subclinical isolates, mostly *agr*-negative, displayed increased invasion capacity, which could facilitate intracellular persistence and immune evasion, leading to chronic infections with mild or absent clinical signs. These differences suggest that therapeutic strategies should consider both adhesion-inhibiting agents for clinical cases and intracellularly active antimicrobials or alternative approaches, such as host-directed therapies, for subclinical infections. Understanding these distinct pathogenic mechanisms could aid in developing targeted interventions to reduce bacterial persistence and mitigate disease progression in bovine mastitis.

A limitation of our study is that, although the observed correlation between the *agr*/MSCRAMM systems and the levels of adhesion and invasion in the strains is relevant, the absence of molecular cloning to validate these associations restricts the interpretation of the results. Cloning specific genes would allow for a more precise analysis of causal relationships, eliminating confounding factors and robustly confirming the direct contribution of these systems to adhesion and invasion.

Since our study did not assess host factors, it is important to consider that variables, such as immune response, host genetics, and the composition of the mammary microbiota, may influence the interaction between pathogens and the host, affecting strain adhesion and invasion. These factors should be considered to better understand pathogen–host interactions and their impact on clinical mastitis outcomes.

## 5. Conclusions

This study reveals that the low incidence of MSCRAMM genes and the absence of *agr* are directly associated with high invasion rates in subclinical isolates, while clinical strains, mainly presenting *agrII* and the presence of several MSCRAMM genes, demonstrate a higher capacity for adhesion, regardless of severity. These findings advance our understanding of pathogen–host interactions, opening the way for more precise and targeted approaches in mastitis control.

## Figures and Tables

**Table 1:** Distribution of *agr* types and MSCRAMM genes in *S. aureus* isolated from cows with clinical (mild and moderate) and subclinical mastitis.

Gene	Clinical Mastitis		Subclinical Mastitis	*p*-Value
	Mild, % (n = 14)	Moderate, % (n = 14)	% (n = 14)	
*agrI*	14.2 (2/14)	14.2 (2/14)	7.1 (1/14)	0.4525
*agrII*	50 (7/14)	42.8 (6/14)	-	<0.0014
*agrIII*	28.5 (4/14)	50 (7/14)	7.1 (1/14)	0.5071
*agr-*	-	-	85.7 (12/14)	<0.0001
*fnbA*	85.7 (12/14)	85.7 (12/14)	14.2(2/14)	<0.0001
*fnbB*	7.1 (1/14)	7.1 (1/14)	-	0.4390
*fib*	92.8 (13/14)	100 (14/14)	7.1 (1/14)	<0.0001
*clfA*	92.8 (13/14)	100 (14/14)	14.2 (2/14)	<0.0001
*clfB*	21.4 (3/14)	21.4 (3/14)	-	0.0718
*cna*	7.1 (1/14)	35.7 (5/14)	-	0.0718
*eno*	92.8 (13/14)	100 (14/14)	100 (14/14)	0.6666
*epbS*	78.5 (11/14)	78.5 (11/14)	14.2 (2/14)	<0.0001

**Table 2:** Relationship between *agr* types (*agrI*, *agrII*, *agrIII*) and some MSCRAMM genes with adhesion and cellular invasion of *S. aureus* isolated from cows with subclinical and clinical mastitis.

Genes	Results	n	Adhesion				Invasion				Adhesion/Invasion Ratio			
			Median	Min	Max	*p-*Value	Median	Min	Max	*p-*Value	Median	Min	Max	*p-*Value
*agrI*	Pos	5	6.00	4.34	6.71	0.2584	4.04	3.45	5.85	0.7871	1.12	0.83	1.95	0.4194
	Neg	37	4.79	2.30	6.96		4.26	2.60	6.36		1.09	0.58	2.40	
*agrII*	Pos	13	6.00	3.51	6.96	0.2948	3.78	2.85	6.00	0.1422	1.26	0.94	2.40	0.0083
	Neg	29	4.77	2.30	6.71		4.93	2.60	6.36		1.03	0.58	1.95	
*agrIII*	Pos	12	5.40	3.34	6.53	0.6986	4.56	2.60	5.91	0.9669	1.12	0.87	1.85	0.4648
	Neg	30	4.78	2.30	6.96		4.23	2.85	6.36		1.06	0.58	2.40	
*agr_negative_*	Pos	12	3.95	2.30	5.81	0.004	4.67	3.23	6.36	0.1234	0.83	0.58	1.03	<0.0001
	Neg	30	6.00	3.34	6.96		3.93	2.60	6.00		1.23	0.83	2.40	
*fnbA*	Pos	26	5.40	3.34	6.96	0.1341	3.93	2.60	6.00	0.2304	1.21	0.83	2.40	0.005
	Neg	16	4.75	2.30	6.61		4.67	3.23	6.36		0.84	0.58	1.74	
*fnbB*	Pos	2	6.63	6.56	6.71	0.048	4.65	3.45	5.85	1	1.53	1.12	1.95	0.2329
	Neg	40	4.78	2.30	6.96		4.23	2.60	6.36		1.09	0.58	2.40	
*fib*	Pos	28	6.13	3.34	6.96	0.0054	3.93	2.60	6.00	0.2367	1.23	0.83	2.40	0.0001
	Neg	14	4.37	2.30	6.00		4.67	3.23	6.36		0.83	0.58	1.74	
*clfA*	Pos	29	6.00	3.34	6.96	0.0175	3.81	2.60	6.00	0.0992	1.22	0.83	2.40	0.0001
	Neg	13	4.32	2.30	6.61		4.93	3.23	6.36		0.83	0.58	1.28	
*clfB*	Pos	6	6.21	3.68	6.59	0.4984	4.01	2.85	5.40	0.3555	1.34	1.01	1.72	0.0888
	Neg	36	4.78	2.30	6.96		4.23	2.60	6.36		1.08	0.58	2.40	
*cna*	Pos	7	5.97	3.74	6.60	0.3944	3.65	2.60	5.78	0.3502	1.23	1.08	1.85	0.0991
	Neg	35	4.77	2.30	6.96		4.26	2.85	6.36		1.07	0.58	2.40	
*eno*	Pos	41	4.79	2.30	6.96	0.6523	4.26	2.60	6.36	0.2081	1.09	0.58	2.40	0.1683
	Neg	1	6.00	6.00	6.00		3.45	3.45	3.45		1.74	1.74	1.74	
*ebpS*	Pos	24	4.81	3.34	6.60	0.7427	3.93	2.60	6.00	0.1622	1.22	0.83	1.85	0.0185
	Neg	18	4.84	2.30	6.96		4.67	2.90	6.36		0.88	0.58	2.40	

## Data Availability

The original contributions presented in the study are included in the article and Supplementary Materials. Further inquiries can be directed to the corresponding author.

## Supplementary Materials

The following supporting information can be downloaded at: https://www.mdpi.com/article/10.3390/vetsci12030270/s1, Table S1: Primers used for the amplification of *agr* cluster and MSCRAMMs genes in *Staphylococcus aureus*. Refs. [26,27] are cited here.
