# Supplementary material for: In Vitro Adhesion and Invasion Rates of Staphylococcus aureus Isolated from Mastitic Cows Are Modulated by the agr System and MSCRAMM Genes
Source: Vet Sci. 2025 Mar 13;12(3):270. doi: 10.3390/vetsci12030270 (PMC11945600; doi:10.3390/vetsci12030270)
Supplement: Supplementary file 1 [file vetsci-12-00270-s001.zip › vetsci-3485682-supplementary.pdf]

Supplementary materials

Table S1: Primers used for the amplification of *agr* cluster and MSCRAMMs genes in *Staphylococcus aureus*

Gene	Primer	Nucleotide sequence	Amplicon size (Bp)	T° melting	Reference
agrI	AGR1-F	ATGCACATGGTGCACATGC	440	55ºC	Shopsin et al., 2003
	AGR1-R	GTCACAAGTACTATAAGCTGCGAT			
agrII	AGR2-F	ATGCACATGGTGCACATGC	572	55ºC	Shopsin et al., 2003
	AGR2-R	GTATTACTAATTGAAAAGTGCCATAGC			
agrIII	AGR3-F	ATGCACATGGTGCACATGC	406	55ºC	Shopsin et al., 2003
	AGR3-R	CTGTTGAAAAAGTCAACTAAAAGCTC			
agrIV	AGR4-F	ATGCACATGGTGCACATGC	588	55ºC	Shopsin et al., 2003
	AGR4-R	CGATAATGCCGTAATAC CCG			
cna	CNA-1	GTCAAGCAGTTATTAACACCAGAC	423	55ºC	Tristan et al., 2003
	CNA-2	AATCAGTAATTGCACTTTGTCCACTG			
eno	ENO-1	ACGTGCAGCAGCTGACT	302	55ºC	Tristan et al., 2003
	ENO-2	CAACAGCATYCTTCAGTACCTTC			
ebpS	EBP-1	CATCCAGAACCAATCGAAGAC	186	55ºC	Tristan et al., 2003
	EBP-2	CTTAACAGTTACATCATCATGTTTATCTTTG			
fnbA	FNBA-1	GTGAAGTTTTAGAAGGTGGAAAGATTAG	643	55ºC	Tristan et al., 2003
	FNBA-2	GCTCTTGTAAGACCATTTTTCTTCAC			
fnbB	FNBB-1	GTAACAGCTAATGGTCGAATTGATACT	524	55ºC	Tristan et al., 2003
	FNBB-2	CAAGTTCGATAGGAGTACTATGTTC			
fiB	FIB-1	CTACAACTACAATTGCCGTCAACAG	404	55ºC	Tristan et al., 2003
	FIB-2	GCTCTTGTAAGACCATTTTCTTCAC			
clfA	CLFA-1	ATTGGCGTGGCTTCAGTGCT	292	55ºC	Tristan et al., 2003
	CLFA-2	CGTTTCTTCCGTAGTTGCATTTG			
clfB	CLFB-1	ACATCAGTAATAGTAGGGGGCAAC	205	55ºC	Tristan et al., 2003
	CLFB-2	TTCGCACTGTTTGTGTTTGCAC			

References

Shopsin, B., B. Mathema, P. Alcabes, B. Said-Salim, G. Lina, A. Mat-suka, J. Martinez, and B. N. Kreiswirth. 2003. Prevalence of agr specificity groups among *Staphylococcus aureus* strains coloniz-ing children and their guardians. J. Clin. Microbiol.41:456–459.https://doi.org/10.1128/JCM.41.1.456-459.2003.

Tristan, A., L. Ying, M. Bes, J. Etienne, F. Vandenesch, and G. Lina.2003. Use of multiplex PCR to identify *Staphylococcus aureus* adhesins involved in human hematogenous infections. J. Clin. Mi-crobiol. 41:4465–4467. https://doi.org/10.1128/JCM.41.9.4465-4467.2003.
